# ﻿Confirming the presence of *Lasiurusfrantzii* (Peters, 1870) (Chiroptera, Vespertilionidae) in South America: more questions than answers

**DOI:** 10.3897/zookeys.1180.105497

**Published:** 2023-09-19

**Authors:** Héctor E. Ramírez-Chaves, Alexandra Cardona-Giraldo, Paula A. Ossa-López, Héctor Fabio Arias Monsalve, Fredy A. Rivera-Páez, Darwin M. Morales-Martínez

**Affiliations:** 1 Grupo de Investigación en Genética, Biodiversidad y Manejo de Ecosistemas (GEBIOME), Departamento de Ciencias Biológicas, Facultad de Ciencias Exactas y Naturales, Universidad de Caldas, Calle 65 No. 26-10, Manizales, Caldas 170004, Colombia; 2 Centro de Museos, Museo de Historia Natural, Universidad de Caldas, Calle 65 No 26-10, Manizales, Caldas, Colombia; 3 Fundación Ecológica Cafetera -FEC-, Km 11 vía al Magdalena, Manizales, Colombia; 4 Museum of Natural Science and Department of Biological Sciences, Louisiana State University, 119 Foster Hall 70803, Baton Rouge, Louisiana, USA

**Keywords:** Andes, bats, Colombia, cryptic, distribution, morphology

## Abstract

The western or desert red bat, *Lasiurusfrantzii*, is a cryptic insectivore species distributed in the Neotropics from Mexico south through Central America to Panama. *L.frantzii* was long considered a subspecies of the red bat, *Lasiurusblossevillii*, but recently it was elevated to full-species status based on genetic information. Here we present evidence of the presence of *L.frantzii* in the Andean Region of Colombia, confirming the species’ presence in South America; the new record, from 3836 m a.s.l., is also the highest elevation known for the species. We suggest that *L.frantzii* might be widely distributed in trans-Andean areas of Colombia, Ecuador, Venezuela, and perhaps Peru and Bolivia. However, a review and exploration of additional morphological traits to identify the species are necessary because of the uncertainty of the distribution of *L.frantzii*.

## ﻿Introduction

The genus *Lasiurus* Gray, 1831 (Chiroptera, Vespertilionidae) comprises 20 species of insectivorous bats distributed in the Americas (Mammal Diversity Database 2023; Simmons and Cirranello 2023). The genus comprises small to medium-sized species with brightly coloured or banded dorsal pelage and a thickly furred dorsal uropatagium (Gardner and Handley 2008; Reid 2009). In recent years, *Lasiurus* has been separated into three distinct genera (Baird et al. 2015, 2017, 2021): *Aeorestes* Fitzinger, 1870, *Dasypterus* Peters, 1870, and *Lasiurus*; however, currently *Aeorestes* and *Dasypterus* are considered to be subgenera (Simmons and Cirranello 2023).

In South America, six of the 11 species belonging to the nominotypical subgenus Lasiurus have been historically documented: *L.arequipae* Málaga, Díaz, Arias & Medina, 2020; *L.atratus* Handley, 1996; *L.blossevillii* (Lesson & Garnot, 1826); *L.castaneus* Handley, 1960; *L.ebenus* Fazzolari-Corrêa, 1994; and *L.varius* Poeppig, 1835. Of these, *L.blossevillii* is considered widely distributed in South America, with records in Colombia, Venezuela, Trinidad and Tobago, Guyana, Suriname, French Guiana, Ecuador (including the Galápagos), Peru, Bolivia, Brazil, Argentina, Paraguay, and Uruguay (Díaz et al. 2021; Mammal Diversity Database 2023). In the northern part of South America (western Colombia and Ecuador and northern Venezuela), Gardner and Handley (2008) included the presence of *L.b.frantzii* (Peters, 1870), which was described from Costa Rica but also inhabits Mexico and several countries in Central America. Recently, *L.frantzii* was elevated to full species by Baird et al. (2015: 1265) who suggested that it “does extend into northern South America while the strictly South American forms would be in a separate species, *L.blossevillii*.” Baird et al. (2015) showed that *L.blossevillii* is easily separated of *L.frantzii* based on the deep divergence of the mtDNA and supposed restricted the distribution of *L.frantzii* to Central America (Mexico to Panama). Previous suggestions that this taxon (as a subspecies of *L.blossevillii*) is present in northern South America were based on morphological and distribution analyses (Handley 1960; Gardner and Handley 2008; Morales-Martínez and Ramírez-Chaves 2015).

A recent study (i.e. Rojas-Herrera et al. 2023) has included *L.frantzii* in northern South America (Colombia, Ecuador, and Venezuela) based on the conclusions of previous works (Gardner and Handley 2008; Baird et al. 2015; Morales-Martínez and Ramírez-Chaves 2015), but there was no information on the limits of the distribution of *L.blossevillii*, which to date is still considered widely distributed in South America (Mammal Diversity Database 2023). Similarly, the altitudinal distribution ranges are unclear for both species. Although *L.blossevillii* has been documented reaching elevations up to 2400 m in Peru (Graham 1983), in Colombia and Ecuador the species is documented up to 2900 (Gardner and Handley 2008; Morales-Martínez and Ramírez-Chaves 2015; Tirira 2017), and it is unclear if that elevation effectively corresponds to records of *L.blossevillii* or *L.frantzii*.

Here, we confirm the presence of *L.frantzii* in Colombia supported by genetic evidence, extend the elevational distribution of this species, and suggest hypotheses on the distribution of *L.frantzii* and *L.blossevillii* in South America.

## ﻿Methods

**Figure 1.**
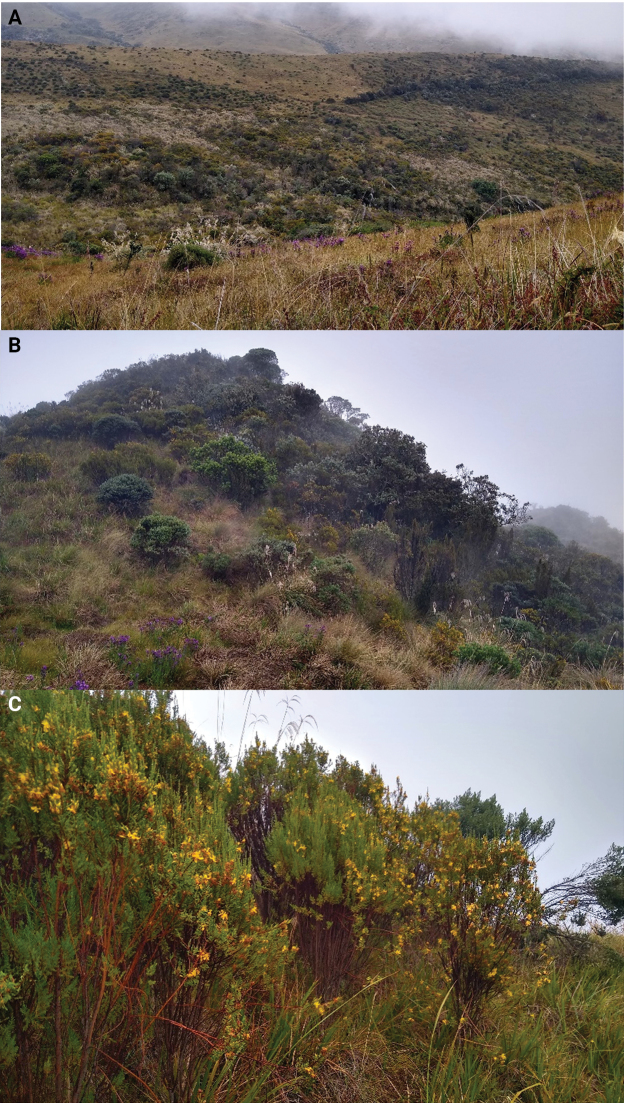
Details of the locality record at the Central Cordillera in Villamaría, Caldas, Colombia **A** Páramo ecosystem dominated by plants of the genus *Espeletia***B** high Andean grassland and shrubland areas **C** the plant of the genus *Hypericum* in which *L.frantzii* was found.

The new record is based on a single female specimen found near a páramo ecosystem, a grassland-shrubland area found at elevations between ~3000 and 5000 m in northern South America to northern Peru (Fig. 1). The locality of the record is the Bosques de La CHEC, Predio Romeral II (4.9454°N, -75.3892°W; 3836 m a.s.l.), municipality of Villamaría, Department of Caldas, in the Central Andes of Colombia. The specimen was found dormant, at the base of a bush of the genus *Hypericum* L. (Clusiaceae) on 1 September 2020.

We collected and deposited the specimen in the Mammal Collection of the Museo de Historia Natural of the Universidad de Caldas (MHN-UCa-M) in Manizales, Colombia. For the morphological characterization we obtained 13 skull and five external measurements (in mm) using digital calipers accurate to 0.01 mm: greatest length of the skull (**GLS**), postorbital constriction (**PC**), least interorbital breadth (**LIB**), zygomatic breadth (**ZB**), braincase breadth (**BB**), palatal length (**PL**), condylo-basal length (**CBL**), mastoid breadth (**MB**), breadth across upper canines (**C-C**), breadth across upper molars (**M-M**), mandibular length (**ML**), maxillary toothrow length (**LMxT**), mandibular toothrow length (**LMdT**), total length (**TL**), tail length (**Tail**), and ear length (**Ear**), hindfoot length (**HF**), and forearm length (**FA**) (Simmons and Voss 1998). We also compared the morphological traits of the new specimen with those from other *Lasiurus* taxa found in South America (Handley 1960; Málaga et al. 2020).

### ﻿Molecular analyses and identification

To confirm the identification of the specimen of *Lasiurus* collected, we extracted genomic DNA from muscle tissues preserved in 96% ethanol. DNA was extracted with a Wizard Genomic DNA Purification kit (Promega Corporation) following the manufacturer’s protocol. We obtained a sequence of the mitochondrial cytochrome-b (cyt-b) gene (896 bp) using the primers LGL765F (Bickham et al. 1995) and LGL766R (Bickham et al. 2004), and the concentrations and the reaction conditions as in Ramírez-Chaves et al. (2021). To complete the data set for comparisons, we gathered 68 homologous sequences of 16 species of *Lasiurus* from GenBank (Suppl. material 1). We used *Myotisriparius* and *Eptesicusfuscus* as outgroups.

We aligned all the sequences of each gene using the default parameters of the Clustal W algorithm in BioEdit v. 7.2.6 software (Hall 1999). We assessed the best-fit evolutionary model using ModelFinder (Kalyaanamoorthy et al. 2017) implemented in IQ-TREE (Nguyen et al. 2015) software specifying the TIM2+F+I+G4 model. We conducted a maximum-likelihood analysis using IQ-TREE (Nguyen et al. 2015) using 20,000 replicates to find the best tree. We used nonparametric SH-aLRT and ultrafast-bootstrap (UFBoot; Hoang et al. 2018) values as the branch support measure. Finally, we used MEGA 7 (Kumar et al. 2015) to calculate average uncorrected *p*-distances with partial deletion, allowing for less than 5% of gaps, missing data, and ambiguous bases, resulting in a new matrix of 800 bp for distance calculations. The percentage of genetic divergence was computed as genetic distance × 100.

### ﻿Distribution hypothesis

Based on information available in the literature (e.g. Handley 1960; Gardner and Handley 2008; Morales-Martínez and Ramírez-Chaves 2015) and our new record, we suggest distribution hypotheses of *L.blossevillii* and *L.frantzii* in South America.

## ﻿Results

**Figure 2.**
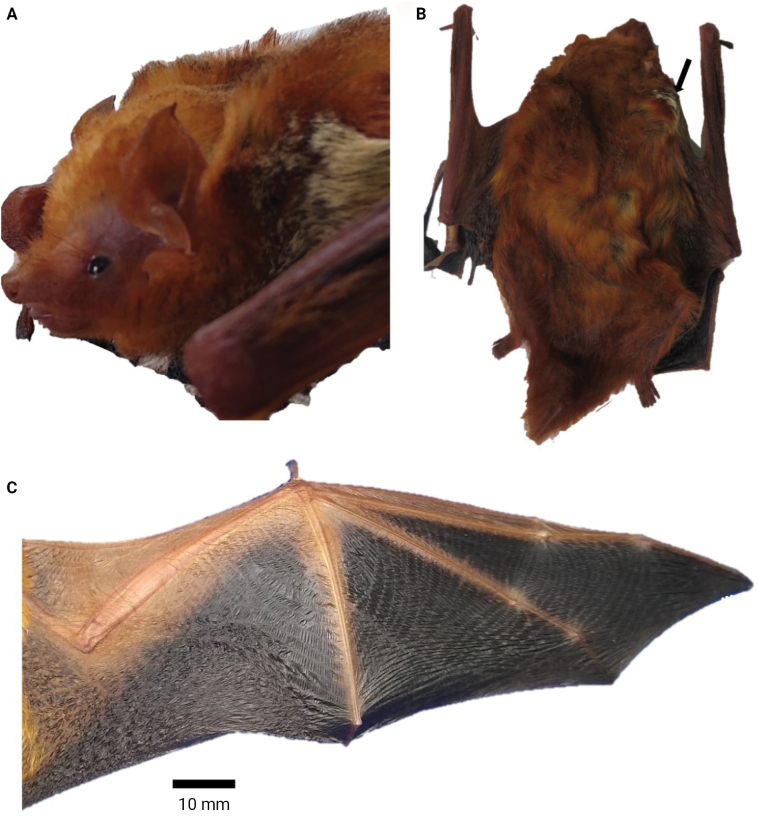
Details of the specimen of *Lasiurusfrantzii* (MHN-UCa-M 3317) from Colombia **A** pinkish face and ears **B** details of the light spot in the shoulders **C** membranes with reddish markings.

**Figure 3.**
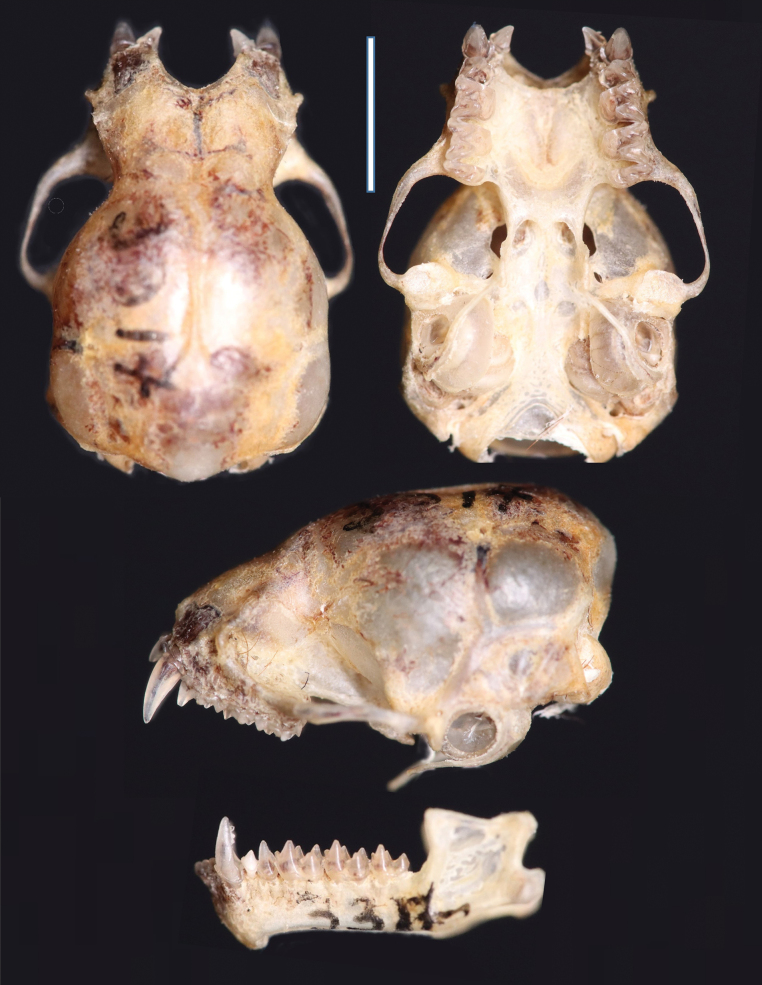
Details of the skull of *Lasiurusfrantzii* (MHN-UCa-M 3317) from Colombia, South America.

The specimen (MHN-UCa-M 3317) was identified as belonging to the subgenus Lasiurus based on the following external and cranial morphological traits: soft and dense fur with bright reddish colouration dorsally, the ventral fur is lighter with paler tones (Fig. 2). The uropatagium is long and with abundant hair of similar colouration to the shoulders. The specimen has morphological traits that resemble those of *Lasiurusblossevillii*, *L.frantzii*, and *L.varius* (Handley 1960; Málaga et al. 2020). MHN-UCa-M 3317 differs from other species of the genus *Lasiurus* (Table 1) by the smaller forearm (< 43 mm *vs* > 44 mm in *L.arequipae*, *L.atratus*, and *L.castaneus*). MHN-UCa-M 3317 is differentiated from *L.varius* by the lighter (pinkish) colouration of the face (blackish in *L.varius*) and wings with lighter colouration near to the sides of forearms and metacarpus (totally black in *L.varius*). MHN-UCa-M 3317 can be differentiated from *L.blossevillii* by the lighter (pinkish) colouration of the face (blackish in *L.blossevillii*), the presence of a patch of lighter hairs on the shoulders (absent in *L.blossevillii*; Málaga et al. 2020), and the dorsum reddish without whitewash (dorsum washed with whitish “frost” in *L.blossevillii*; Handley 1960). MHN-UCa-M 3317 also showed the presence of moderate fat in the pelvis and near the axillary mammary glands. Cranially MHN-UCa-M 3317 exhibited two upper premolars per ramus (Fig. 3), similar to other species of red bats.

**Table 1.** External and cranial measurements (mm) and weight (g) of the confirmed record of *Lasiurusfrantzii* in South America. Measurements of *L.varius* and *L.blossevillii* taken from Málaga et al. (2020). Numbers include mean ± SD, ranges in parentheses, and sample size.

Measurement	*L.frantzii*MHN-UCa-M 3317 female	*L.varius*	*L.blossevillii*
Total length (TL)	112	108.6 ± 4.87 (105.0–118.0) 7	100.6 ± 5.65 (92.0–112.0) 10
Tail length (tail)	55.5	52.1 ± 4.67 (44.0–58.0) 7	47.9 ± 2.77 (44.5–52.0) 10
Hindfoot	8.5	7.3 ± 1.44 (6.0–10.0) 7	8.0 ± 0.75 (7.0–9.0) 8
Ear length (ear)	11.55	11.8 ± 1.84 (9.0–13.9) 7	10.3 ± 1.16 (8.0–11.6) 10
Forearm	42.91	40.6 ± 0.88 (39.9–42.1) 7	39.3 ± 1.22 (37.7–41.3) 12
W	11 g	0.2 ± 1.06 (9.5–11.0) 2	8.2 ± 1.36 (6.0–10.0) 6
GLS	12.66	13.0 ± 0.21 (12.8–13.4) 7	11.8 ± 0.38 (11.3–12.5) 10
PC	4.44	4.5 ± 0.13 (4.3–4.7) 7	4.2 ± 0.14 (4.1–4.5) 11
LIB	5.64	6.0 ± 0.08 (5.9–6.1) 7	5.3 ± 0.18 (4.9–5.5) 11
ZB	9.24	9.7 ± 0.10 (9.6–9.8) 7	8.7 ± 0.38 (8.3–9.4) 7
BB	7.59	7.7 ± 0.19 (7.5–8.0) 7	7.3 ± 0.38 (6.6–7.8) 11
PL	4.07	5.5 ± 0.20 (5.3–5.9) 6	4.8 ± 0.26 (4.4–5.0) 7
CBL	12.07	12.6 ± 0.24 (12.2–12.9) 7	11.1 ± 0.47 (10.3–11.8) 10
MB	7.75	7.8 ± 0.39 (7.0–8.2) 7	7.4 ± 0.22 (7.1–7.9) 10
C-C	5.29	5.2 ± 0.16 (5.0–5.4) 7	4.4 ± 0.18 (4.1–4.7) 10
M-M	5.79	6.3 ± 0.13 (6.1–6.5) 7	5.3 ± 0.28 (4.9–5.7) 11
LM	9.39	9.8 ± 0.09 (9.7–10.0) 7	8.7 ± 0.29 (8.4–9.4) 9
LMxT	4.22	4.6 ± 0.07 (4.5–4.7) 7	3.9 ± 0.14 (3.6–4.1) 11
LMdT	5.13	5.3 ± 0.05 (5.3–5.4) 7	4.6 ± 0.10 (4.5–4.8) 10

**Figure 4.**
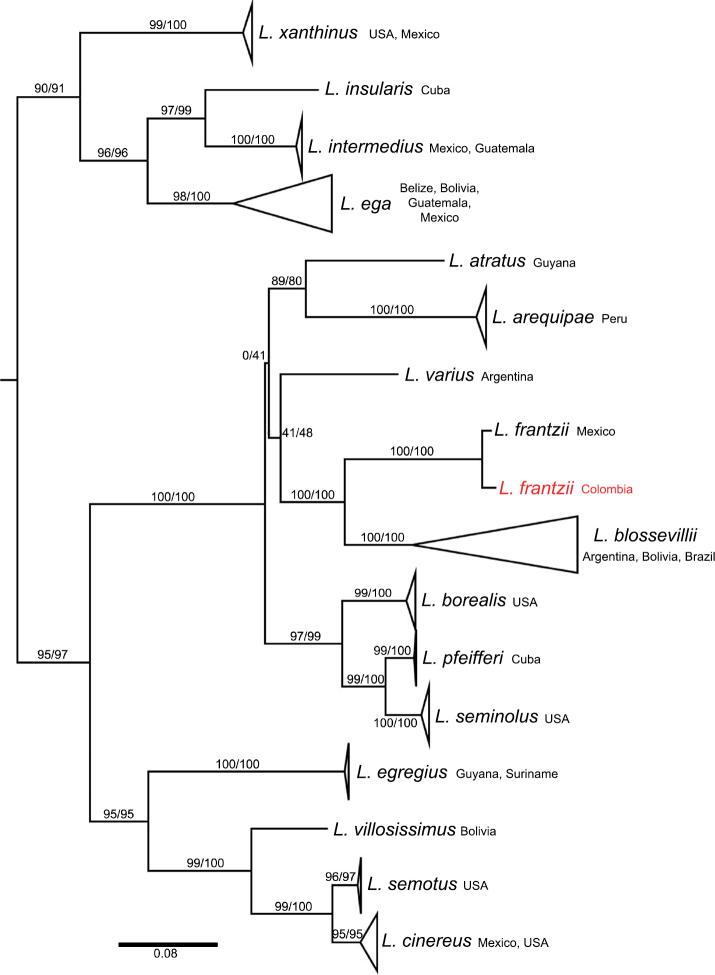
Maximum-likelihood gene tree of *Lasiurus* based on the cyt-b gene. The values of the branches indicate the maximum-likelihood nonparametric inference (SH-aLRT) and ultrafast (UFBoot) bootstrap values, respectively.

Results of the molecular identification based on cyt-b showed that our specimen is in the same clade as the sequence of *L.frantzii* from Mexico with strong support (SH-aLRT = 100; UFBoot = 100). This clade is sister to *L.blossevillii* (SH-aLRT = 100; UFBoot = 100); the clade is recovered with strong support (SH-aLRT = 100; UFBoot = 100; Fig. 4). This close relationship confirms the identification of MHN-UCa-M 3317 as *L.frantzii*. The genetic distance between the specimen MHN-UCa-M 3317 from Colombia and *L.frantzii* from Mexico is 1.85%. Genetic distances of *L.frantzii* clade versus other species are within 12.71% with its sister clade *L.blossevillii* and 20.24% with *L.egregius* (Table 2).

**Table 2.** Uncorrected *p*-distances in % for the cyt-b gene between species of *Lasiurus* (sensu lato, including taxa of the (sub)genera *Aeorestes* and *Dasypterus*). Bold values in the diagonal represent the distances within taxa.

	1	2	3	4	5	6	7	8	9	10	11	12	13	14	15	16
1. *L.arequipae*	**0.78**															
2. *L.atratus*	15.59	**NA**														
3. *L.blossevillii*	16.12	15.38	**3.5**													
4. *L.borealis*	14.40	14.34	14.63	**0.79**												
5. *L.cinereus*	18.23	18.02	19.14	17.23	**0.52**											
6. *L.ega*	20.49	19.23	20.92	19.08	18.54	**7.20**										
7. *L.egregius*	18.97	18.38	18.75	17.70	15.64	16.65	**0.25**									
8. *L.frantzii*	16.06	14.50	12.71	15.41	19.86	21.34	20.24	**1.85**								
9. *L.insularis*	18.33	17.88	18.33	19.05	18.42	15.35	16.75	18.70	**NA**							
10. *L.intermedius*	19.61	19.06	19.69	20.10	18.00	13.51	15.69	19.82	10.19	**0.50**						
11. *L.pfeifferi*	15.97	15.55	13.21	8.58	18.39	19.06	17.88	15.67	18.24	19.30	**0.27**					
12. *L.seminolus*	16.30	15.35	14.66	9.21	17.58	18.15	18.48	15.77	18.55	19.34	4.79	**0.53**				
13. *L.semotus*	19.70	17.68	19.88	18.30	4.52	17.98	16.45	20.16	17.83	17.99	19.22	17.98	**0.15**			
14. *L.varius*	15.27	14.63	12.79	13.29	17.36	18.90	18.13	14.65	18.63	19.69	13.94	13.68	17.15	**NA**		
15. *L.villosissimus*	18.72	17.50	16.63	16.51	9.36	18.33	14.50	18.42	17.63	17.69	17.81	17.75	9.83	15.88	**NA**	
16. *L.xanthinus*	19.33	18.89	18.80	18.41	16.79	15.79	15.89	20.07	14.68	15.33	18.67	18.98	16.60	18.54	16.32	**0.25**

**Figure 5.**
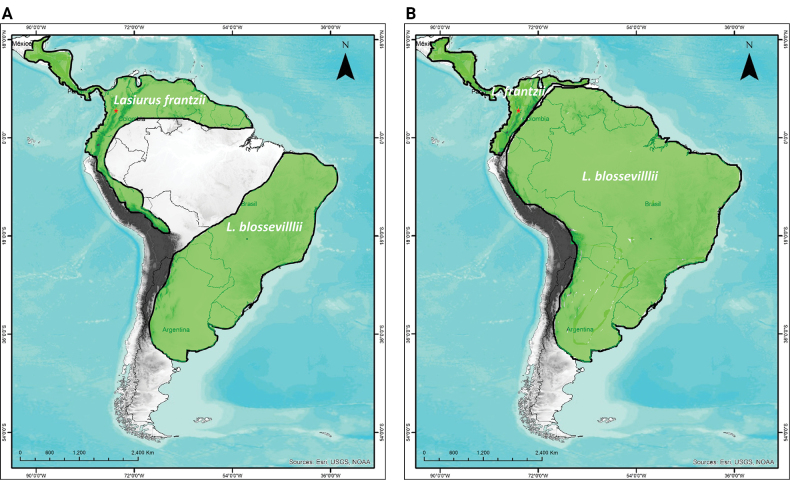
Two hypotheses on the distribution of *Lasiurusfrantzii* and *L.blossevillii* in South America **A** where the Amazon Forest acts as a barrier and *L.blossevillii* is restricted to the Southern Cone **B** where both species are present in northern South America.

Based on information available in the literature (e.g. Handley 1960; Gardner and Handley 2008; Morales-Martínez and Ramírez-Chaves 2015) and our new record, *L.frantzii* reaches northern South America and likely extends south as far as the Andes of Peru and Bolivia (Fig. 5).

## ﻿Discussion

Our results corroborate the presence of *Lasiurusfrantzii* in South America, as has been previously suggested (Gardner and Handley 2008; Morales-Martínez and Ramírez-Chaves 2015), and genetic analysis confirms that the species should be valid. However, the distributions of *L.frantzii* and *L.blossevillii* in northern South America are uncertain. One of the hypotheses is that the Amazon forest limits the distribution of *L.frantzii* (see Gardner and Handley 2008; Morales-Martínez and Ramírez-Chaves 2015), and therefore the species would be widely distributed in the Andean cordillera and cis-Andean localities in northern South America (Handley 1960), including likely records from urban areas in Ecuador (see Nivelo-Villavicencio et al. 2019), Colombia (Morales-Martínez and Ramírez-Chaves 2015), Venezuela (Gardner and Handley 2008); it might reach Peru and Bolivia (Fig. 5). Under this scenario, *L.blossevillii* should represent the records in the Southern Cone (Fig. 5). A second hypothesis is that *L.frantzii* in South America would be restricted to northern and western Colombia, northern Venezuela, and western Ecuador (Gardner and Handley 2008). In this context, both *L.frantzii* and *L.blossevillii* will occur in northern South America, but the Andes serve as a barrier to these species. In any case, our record challenges the previous distribution hypothesis for both taxa (e.g. Shump and Shump 1982; Gardner and Handley 2008) and opens questions about their distribution.

Genetic analyses can help to clarify the identification of eastern Andean populations of *L.blossevillii*, but the geographic coverage is still partial. All genetic samples of *L.blossevillii* available in GenBank come from localities south of the Amazon Basin (Argentina, Bolivia, and Brazil) and represent the nominal *L.blossevillii* (type locality: “la rivière de la Plata,” Buenos Aires, Argentina). No sequences are available from north of the Amazon Basin. Therefore, further studies using genetics (Baird et al. 2015) and morphological characters (Handley 1960; Málaga et al. 2020) with broad geographical sampling are necessary to clarify the distribution of *L.frantzii* and *L.blossevillii*.

To the best of our knowledge, the new record of *L.frantzii* from Colombia, at 3836 m a.s.l., is the highest documented for any *Lasiurus* (sensu stricto) species and is likely the highest elevation record for any red bat in Colombia. Other high-elevation records of bats in South America belong to vespertilionid species such as *Histiotus* in Ecuador and Peru; the highest elevational records reach 4000 m (Graham 1983; Rodríguez-Posada et al. 2021). However, key aspects of the biology of these species, such as the ecology and metabolic characteristics, have been little studied.

Finally, the taxonomy of vespertilionid bats has been assessed recently for all the Neotropical genera (Baird et al. 2008, 2021; Larsen et al. 2012; Carrión-Bonilla and Cook 2020). However, most of studies do not include information from Colombia despite this country’s biogeographical complexity (but see Ramírez-Chaves et al. 2021, 2023; Rodríguez-Posada et al. 2021); this makes the delimitation of species’ distribution, the identification of the populations of both sides of the Andes, and the formulation of biogeographical hypothesis challenging. For example, several reviews of the widespread genus such as *Myotis* included no genetic samples from Colombia (e.g. Larsen et al. 2012; Carrión-Bonilla and Cook 2020), and our sequence is the first available for *Lasiurus* (sensu lato) from the country.

## ﻿Additional information

### Author ORCIDs

Héctor E. Ramírez-Chaves https://orcid.org/0000-0002-2454-9482

Alexandra Cardona-Giraldo https://orcid.org/0000-0002-7534-994X

Paula A. Ossa-López https://orcid.org/0000-0002-9079-4988

Héctor Fabio Arias Monsalve https://orcid.org/0000-0003-0783-2611

Fredy A. Rivera-Páez https://orcid.org/0000-0001-8048-5818

Darwin M. Morales-Martínez https://orcid.org/0000-0001-5786-4107

### Data availability

All of the data that support the findings of this study are available in the main text or Supplementary Information.

## Supplementary materials

Supplementary material 1 Voucher number and Genbank accession codes of the sequences of *Lasiurus* used in this study Data type docx Explanation note The new sequence from Colombia is highlighted in bold. MHN-UCa: Museo de Historia Natural, Universidad de Caldas, Colombia. https://binary.pensoft.net/file/908274This dataset is made available under the Open Database License (http://opendatacommons.org/licenses/odbl/1.0/). The Open Database License (ODbL) is a license agreement intended to allow users to freely share, modify, and use this Dataset while maintaining this same freedom for others, provided that the original source and author(s) are credited.Héctor E. Ramírez-Chaves, Alexandra Cardona-Giraldo, Paula A. Ossa-López, Héctor Fabio Arias Monsalve, Fredy A. Rivera-Páez, Darwin M. Morales-Martínez
