# Supplementary material for: ﻿Confirming the presence of Lasiurusfrantzii (Peters, 1870) (Chiroptera, Vespertilionidae) in South America: more questions than answers
Source: Zookeys. 2023 Sep 19;1180:145–57. doi: 10.3897/zookeys.1180.105497 (PMC10843333; doi:10.3897/zookeys.1180.105497)
Supplement: Supplementary material 1 — Voucher number and Genbank accession codes of the sequences of Lasiurus used in this study [file zookeys-1180-145_article-105497__-s001.docx]

**Table S1.** Voucher number and Genbank accession codes of the sequences of *Lasiurus* used in this study. The new sequence from Colombia is highlighted in bold. MHN-UCa: Museo de Historia Natural, Universidad de Caldas, Colombia.

| Species | Voucher number | GenBank accession | Locality |
| --- | --- | --- | --- |
| *Lasiurus arequipae* | MUSA21058 | MN523651 | Peru: Arequipa |
| *Lasiurus arequipae* | MUSM52933 | OK030950 | Peru: Tacna |
| *Lasiurus atratus* | F54400 | KP341704 | Guyana |
| *Lasiurus blossevillii* | MSB63295 | KC747683 | Bolivia: Santa Cruz |
| *Lasiurus blossevillii* | AK13464 | KP341705 | Argentina |
| *Lasiurus blossevillii* | MVZAD522 | AF376838 | Brazil: Paraná |
| *Lasiurus borealis* | AK21072 | KP341709 | USA: Kansas |
| *Lasiurus borealis* | AK10319 | KP341708 | No data |
| *Lasiurus borealis* | AK21073 | KP341710 | USA: Kansas |
| *Lasiurus borealis* | AK7214 | MF990028 | USA |
| *Lasiurus borealis* | LSUMZM8125 | KC747684 | USA: Louisiana |
| *Lasiurus cinereus* | BPBM185003 | KP341721 | USA: Hawaii |
| *Lasiurus cinereus* | AK11013 | KP341712 | Mexico: Querétaro |
| *Lasiurus cinereus* | ASK3520 | KP341718 | USA: Texas |
| *Lasiurus cinereus* | NK9250 | KP341738 | Mexico: Sonora |
| *Lasiurus cinereus* | ASK1079 | KP341717 | USA: Texas |
| *Lasiurus cinereus* | BPBM185541 | KP341726 | USA: Hawaii |
| *Lasiurus cinereus* | NK3580 | KP341730 | USA: New Mexico |
| *Lasiurus cinereus* | AK11210 | KP341715 | Mexico: Querétaro |
| *Lasiurus cinereus* | NK08096 | KP341736 | Mexico: Baja California |
| *Lasiurus cinereus* | AK11014 | KP341713 | Mexico: Querétaro |
| *Lasiurus cinereus* | TK78926 | KC747685 | USA: Texas |
| *Lasiurus cinereus* | NK3599 | KP341731 | USA: New Mexico |
| *Lasiurus cinereus* | NK6564 | KP341735 | Mexico: Sonora |
| *Lasiurus cinereus* | NK9191 | KP341737 | USA: New Mexico |
| *Lasiurus cinereus* | NK9273 | KP341739 | Mexico: Sonora |
| *Lasiurus cinereus* | AK11212 | KP341716 | Mexico: Querétaro |
| *Lasiurus cinereus* | NK3563 | KP341729 | USA: New Mexico |
| *Lasiurus cinereus* | NK3625 | KP341732 | USA: New Mexico |
| *Lasiurus cinereus* | AK11097 | KP341714 | Mexico: Querétaro |
| *Lasiurus cinereus* | BPBM185539 | KP341725 | USA: Hawaii |
| *Lasiurus cinereus* | NK3562 | KP341728 | USA: New Mexico |
| *Lasiurus cinereus* | NK3627 | KP341733 | USA: New Mexico |
| *Lasiurus cinereus* | AK11006 | KP341711 | Mexico: Querétaro |
| *Lasiurus cinereus* | NK3642 | KP341734 | USA: New Mexico |
| *Lasiurus ega* | NK12302 | KP341742 | Bolivia |
| *Lasiurus ega* | NK15304 | KP341743 | Bolivia |
| *Lasiurus ega* | AK01635 | KP341740 | Mexico: Tamaulipas |
| *Lasiurus ega* | AK07693 | KP341741 | Belize |
| *Lasiurus ega* | SP12622 | KP341744 | Guatemala |
| *Lasiurus egregius* | F54554 | KP341745 | Guyana |
| *Lasiurus egregius* | F54845 | KP341746 | Suriname |
| *Lasiurus frantzii* | AK11119 | MF990031 | Mexico: Tamaulipas |
| ***Lasiurus frantzii*** | **MHN-UCa 3317** | **OR474549** | **Colombia: Caldas** |
| *Lasiurus insularis* | TK32049 | KP341747 | Cuba |
| *Lasiurus intermedius* | LSUMZM352 | KC747687 | Mexico: Michoacan |
| *Lasiurus intermedius* | ASK0422 | KP341748 | Guatemala |
| *Lasiurus pfeifferi* | TK32016 | KP341749 | Cuba |
| *Lasiurus pfeifferi* | TK32029 | KP341750 | Cuba |
| *Lasiurus pfeifferi* | TK32056 | MF990029 | Cuba |
| *Lasiurus seminolus* | AK21348 | KP341752 | USA: Texas |
| *Lasiurus seminolus* | AK01565 | KP341751 | USA: Texas |
| *Lasiurus seminolus* | LSUMZM8970 | KC747688 | USA: Louisiana |
| *Lasiurus seminolus* | AK06914 | KP341753 | USA: Texas |
| *Lasiurus seminolus* | AK10354 | MF990030 | USA: Texas |
| *Lasiurus semotus* | BPBM185479 | KP341723 | USA: Hawaii |
| *Lasiurus semotus* | BPBM185245 | KP341722 | USA: Hawaii |
| *Lasiurus semotus* | BPBM185538 | KP341724 | USA: Hawaii |
| *Lasiurus semotus* | BPBM178452 | KP341719 | USA: Hawaii |
| *Lasiurus semotus* | BPBM178453 | KP341720 | USA: Hawaii |
| *Lasiurus varius* | AK16070 | KP341756 | Argentina |
| *Lasiurus villosissimus* | NK11502 | KP341727 | Bolivia |
| *Lasiurus xanthinus* | TTU78296 | KC747686 | USA: Texas |
| *Lasiurus xanthinus* | AK21099 | AF369546 | USA: Texas |
| *Lasiurus xanthinus* | NK3579 | AF369547 | USA: New Mexico |
| *Lasiurus xanthinus* | NK11103 | KP341757 | USA: New Mexico |
| *Lasiurus xanthinus* | NM54959 | AF369549 | Mexico: Sonora |
| *Lasiurus xanthinus* | NK11102 | AF369548 | Mexico: Sonora |
| *Lasiurus xanthinus* | MSB45880 | KC747689 | USA: New Mexico |
